# Transcriptome data combined with two-sample Mendelian randomization reveal *IRF1* and *PRKD1* as UPR-related key regulators in intervertebral disc degeneration

**DOI:** 10.1097/MD.0000000000048213

**Published:** 2026-04-03

**Authors:** Feng Zheng, Jiawei Fu, Zhilei Hu, Menglin Luo, Wenbo Yue, Yulin Ma, Chao Liu, Chenhao Liu

**Affiliations:** a Department of Orthopedics, Qinghai Provincial People’s Hospital, Xining, Qinghai, China; b Department of Orthopedics, The Second Affiliated Hospital of Army Medical University, Chongqing, China; c Department of Laboratory, Chongqing Xiqu Hospital, Chongqing, China.

**Keywords:** intervertebral disc degeneration, *IRF1*, *PRKD1*, two-sample Mendelian randomization, unfolded protein response

## Abstract

Intervertebral disc degeneration (IDD) is a leading cause of chronic low back pain, yet the molecular mechanisms driving its progression remain incompletely defined. Emerging evidence suggests that endoplasmic reticulum stress and the unfolded protein response (UPR) may play critical roles in disc cell dysfunction. Transcriptomic datasets (GSE70362 and GSE56081) from human nucleus pulposus tissues were integrated to identify UPR-related differentially expressed genes. Functional enrichment, gene set enrichment analysis, and gene set variation analysis analyses were performed to explore associated biological pathways. Protein–protein interaction networks, transcription factor–target predictions, and immune infiltration analyses were conducted. Two-sample Mendelian randomization was applied to assess potential causal relationships between UPR-related genes and IDD. Key genes were further validated using independent datasets and single-cell RNA sequencing data. A total of 26 UPR-related differentially expressed genes were identified, and protein–protein interaction network analysis with hub gene screening highlighted *IRF1*, *PRKD1*, *CCND1*, and *PDLIM1* as central regulators. Two-sample Mendelian randomization confirmed the causal associations of *IRF1* and *PRKD1* with IDD risk. Immune infiltration analysis revealed significant correlations between UPR-related genes and the activity of macrophages and T cells. Single-cell RNA sequencing further validated the differential expression and cell-type specificity of *IRF1* and *PRKD1* in degenerated nucleus pulposus tissues. This integrative multi-omics and Mendelian randomization study identifies *IRF1* and *PRKD1* as UPR-related drivers of IDD. These findings provide potential biomarkers and therapeutic targets for IDD management.

## 1. Introduction

Intervertebral disc degeneration (IDD) is a leading cause of chronic low back pain, which severely impairs quality of life and imposes a substantial socioeconomic burden worldwide.^[1,2]^ The pathological hallmarks of IDD include extracellular matrix degradation, loss of disc height, nucleus pulposus (NP) cell apoptosis, annulus fibrosus rupture, and endplate calcification.^[3,4]^ Multiple factors contribute to IDD progression, including aging, genetic susceptibility, mechanical overload, metabolic disorders, and inflammatory responses.^[5,6]^ Current treatment strategies, such as analgesics, physiotherapy, and surgical interventions, can relieve symptoms but fail to halt or reverse the degenerative process.^[7,8]^ Therefore, it is important to investigate the mechanisms of IDD and explore potential key regulatory genes for the clinical treatment of this disease.^[9]^

The unfolded protein response (UPR) is a highly conserved cellular adaptive mechanism activated in response to endoplasmic reticulum stress. By restoring protein homeostasis, the UPR determines cell survival or apoptosis under stress conditions.^[10–12]^ Increasing evidence indicates that aberrant UPR activation contributes to IDD pathogenesis.^[13]^ It was demonstrated that tumor necrosis factor α (TNF-α) induced apoptosis in rat NP cells via activation of the UPR, while promoting proliferation of surviving cells,^[14,15]^ whereas pretreatment with UPR inhibitors attenuated TNF-α-induced cell proliferation and exacerbated NP cell apoptosis.^[16,17]^ This suggests that TNF-α can exert antiapoptotic, pro-survival, and proliferative effects in NP cells via UPR under inflammatory stimuli, thereby alleviating the inflammation-associated IDD process. Another study showed that the PERK-ATF4 pathway and its downstream molecule common name for a transcription factor were upregulated in IDD patients with different degrees of degeneration and in rat fibroblast tissues, and the transcription of inflammatory factors in fibroblasts was inhibited by blocking the PERK pathway, which suggests that the PERK pathway in the UPR plays an important role in alleviating inflammation in fibroblasts, and offers a potential therapeutic direction for the treatment of inflammation-mediated IDD.^[16]^ This suggests that the PERK pathway in the UPR plays an important role in alleviating the inflammatory response in fibroblasts, providing a potential therapeutic direction for inflammation-mediated IDD.^[10,18]^ all of which are critical events in disc degeneration. Although UPRhas been implicated in IDD, its specific regulatory genes and causal roles remain largely unclear. Thus, elucidating the contribution of UPR-related genes to IDD may provide novel insights into disease mechanisms and therapeutic strategies.

Mendelian randomization (MR) is a genetic epidemiology approach that leverages genetic variants as instrumental variables to infer causal relationships between exposures and outcomes, thereby minimizing confounding and reverse causation.^[19–21]^ MR has been increasingly applied in musculoskeletal research, including IVDD. For example, a recent bidirectional MR analysis revealed causal associations between cerebrospinal fluid metabolites and IVDD risk, suggesting that altered metabolic profiles may actively contribute to disc degeneration. Another two-step MR study demonstrated that gut microbiota influence IVDD development, with blood metabolites acting as partial mediators of this effect.^[22,23]^ Furthermore, MR investigations of molecular markers of biological aging found that granulocyte proportions and telomere length are causally linked to IVDD, highlighting the role of systemic aging processes in disc pathology.^[24]^ Despite these advances, few studies have examined the causal contributions of unfolded protein response-related genes (UPRGs)to IDD. Applying MR in this context provides a robust framework to identify key regulators of disc degeneration with high translational relevance. However, few studies have explored the causal effects of UPR-related genes on IDD using MR analysis.

In this study, we integrated transcriptomic data from human NP tissues with UPR-related gene sets to identify differentially expressed genes (DEGs) and their enriched biological pathways. Protein–protein interaction (PPI) networks, transcription factor (TF)–target relationships, and immune infiltration patterns were systematically analyzed. MR was further employed to assess the causal effects of candidate UPR-related genes on IDD risk. This multi-omics and MR approach highlights potential biomarkers and therapeutic targets, providing novel mechanistic insights into the role of UPR in IDD progression.

## 2. Materials and methods

### 2.1. Data acquisition and processing

The GSE70362 dataset (platform GPL17810) was downloaded from the gene expression omnibus (GEO) database (https://www.ncbi.nlm.nih.gov/geo/) and used as the training set. It included 16 IDD NP tissue samples and 8 control NP samples (microarray sequencing). Based on Thompson grading, grades I–II were defined as controls and grades III–V as IDD samples. Differential expression analysis was performed using the R package limma.

UPRGs were retrieved from MSigDB, including 3 gene sets: HALLMARK_UNFOLDED_PROTEIN_RESPONSE (113 genes), REACTOME_UNFOLDED_PROTEIN_RESPONSE_UPR (93 genes), and WP_UNFOLDED_PROTEIN_RESPONSE (24 genes). After merging and de-duplication, 168 UPRGs were obtained. The Wilcoxon function of the R package “rstatix” was used to analyze the differential UPRGs in the IDD and control groups of the training set, and the 21 differential UPRGs (d-UPRGs) were used as the background gene set. The single sample gene set enrichment analysis (ssGSEA) algorithm of the R package “GSVA” was used to calculate the ssGSEA scores of the UPRGs for all the samples in the training set. The ssGSEA algorithm of the R package “ssGSEA” was used to calculate the ssGSEA scores of the UPRGs. The Wilcoxon function of the R package “rstatix” was used to analyze the differences in the ssGSEA scores of UPRGs in the IDD and Control groups. The IDD samples of the training set were divided into high and low scoring groups according to the median score of the ssGSEA scores of the UPRGs (median score = 6.516). In the IDD samples of the training set, differential expression analysis was performed on the ssGSEA score groups of high and low UPRGs using the R package “limma” to analyze the DEGs between the high and low-score groups.

The GSE56081 dataset (platform GPL15314), comprising 5 IDD and 5 control NP tissue samples (microarray sequencing), was used as an independent validation dataset.

### 2.2. PPI network construction

Candidate genes were submitted to the STRING database (http://www.string-db.org/, interaction score > 0.4) to construct a PPI network. The results were imported into Cytoscape for visualization.

### 2.3. Two-sample Mendelian randomization analysis

To explore the potential causal effects of candidate genes on IDD, a two-sample MR analysis was conducted using the R package “Two Sample MR.” Single nucleotide polymorphisms (SNPs) significantly associated with candidate genes (*P* < 5 × 10^−8^) were selected as instrumental variables and subjected to linkage disequilibrium pruning (*r*^2^ < 0.001, 10 kb window), with weak instruments excluded by retaining only SNPs with *F*-statistics > 10. Harmonization of exposure and outcome datasets was performed to align effect alleles, and only exposures with at least 3 valid IVs were included in the analysis. Causal effects were primarily estimated using the inverse variance weighted (IVW) method, while MR-Egger regression, weighted median, simple mode, and weighted mode approaches were applied as complementary strategies to ensure robustness. The reliability of causal estimates was further evaluated through a series of sensitivity analyses, including MR-Egger intercept for horizontal pleiotropy, and leave-one-out analysis to assess the influence of individual variants. In addition, the Steiger test was used to confirm the directionality of associations and rule out reverse causality. Only genes consistently supported across multiple methods and sensitivity analyses were considered to have robust causal associations with IDD.

### 2.4. Machine learning-based feature selection

First, least absolute shrinkage and selection operator (LASSO) regression was performed using the “glmnet” R package, which applies a penalty to shrink the regression coefficients of irrelevant or redundant variables towards zero, thereby retaining only the genes with the strongest explanatory power. The optimal penalty parameter (λ) was determined through 10-fold cross-validation to minimize the prediction error, ensuring that the retained features captured the most stable and representative signals. In parallel, the support vector machine–recursive feature elimination (SVM-RFE) algorithm, implemented with the “caret” package, was used to iteratively remove the least significant features while retraining the model at each step, ultimately yielding the set of genes that provided the highest classification accuracy in distinguishing IDD samples from controls. By integrating the results of both algorithms, overlapping genes were considered as the final candidate biomarkers to reduce bias from individual methods and improve reliability.

### 2.5. Functional enrichment analysis

Functional enrichment analyses, including gene ontology (GO), Kyoto Encyclopedia of genes and genomes (KEGG), gene set enrichment analysis (GSEA), and gene set variation analysis (GSVA), were performed to explore the biological roles of key UPR-related genes. GO and KEGG enrichment analyses were conducted using the clusterProfiler R package, which allowed systematic annotation and pathway identification for DEGs. GSEA was implemented with clusterProfiler in combination with reference gene sets obtained from the msigdbr database, enabling evaluation of pathway-level enrichment for individual genes based on ranked gene lists. GSVA, performed using the GSVA R package, provided a nonparametric, sample-wise estimation of pathway activity, converting gene-level expression data into pathway-level enrichment scores. Differences in pathway activity between IDD and control groups were further assessed using the limma package.

### 2.6. Gene function and localization analyses

Subcellular localization was predicted using mRNALocater, and chromosomal positions were visualized with RCircos. Functionally related genes were predicted with GeneMANIA, and gene–gene correlation was assessed with the Spearman method (R package psych).

### 2.7. Immune infiltration analysis

Immune infiltration was analyzed using the ssGSEA algorithm implemented in the GSVA R package to calculate enrichment scores for 28 immune cell types in the training set. Differences in immune cell enrichment between IDD and control samples were assessed with the Wilcoxon test via the rstatix package. Spearman correlation between key genes and differentially enriched immune cells was calculated using the psych R package, with correlation strength interpreted based on |r| values. Additionally, the HTA database was queried to examine RNA and protein expression of key genes across various immune cell types, providing a reference for gene-level immune cell associations.

### 2.8. Regulatory network construction and drug prediction

TFs potentially regulating the key genes (*IRF1*, *PRKD1*) were predicted using the TRRUST database on the miRNet platform (https://www.mirnet.ca/miRNet/home.xhtml). Predicted TF–gene interactions were visualized using Cytoscape. microRNA (miRNAs) targeting the key genes were obtained from the NetworkAnalyst platform (https://www.networkanalyst.ca/NetworkAnalyst/), while miRNA– long non-coding RNA (lncRNA) interactions were predicted using the miRNet (https://www.mirnet.ca/miRNet/home.xhtml), StarBase (https://rnasysu.com/encori/), and http://c1.accurascience.com/miRecords/ DIANA-LncBase (https://diana.e-ce.uth.gr/lncbasev3) databases. The intersection of predicted lncRNAs across the 3 databases was used to construct a lncRNA–miRNA–mRNA regulatory network, which was mapped using Cytoscape. Potential drugs interacting with the key genes were predicted via the DGIdb database (https://www.dgidb.org/)

### 2.9. Single-cell RNA sequencing (scRNA-seq) analysis

Single-cell RNA sequencing data from the GSE199866 dataset were analyzed using the Seurat R package (v4.3.0). Cells expressing fewer than 200 genes, genes detected in fewer than 3 cells, cells with nFeature_RNA < 500 or > 5000, nCount_RNA > 30,000, or mitochondrial gene percentage >5% were filtered out. Batch effects were removed using the FindIntegrationAnchors and IntegrateData functions. Data normalization was performed with NormalizeData, and the top 2000 highly variable genes were identified using FindVariableFeatures. The data were scaled using ScaleData, and dimensionality reduction and clustering were performed with UMAP, setting resolution to 0.6. Monocle (v2.26.0) was applied to perform pseudotime trajectory analysis of key genes, reconstructing cell differentiation paths.

## 3. Results

### 3.1. Identification of candidate genes associated with UPRGs in IVDD

**Figure 1.**
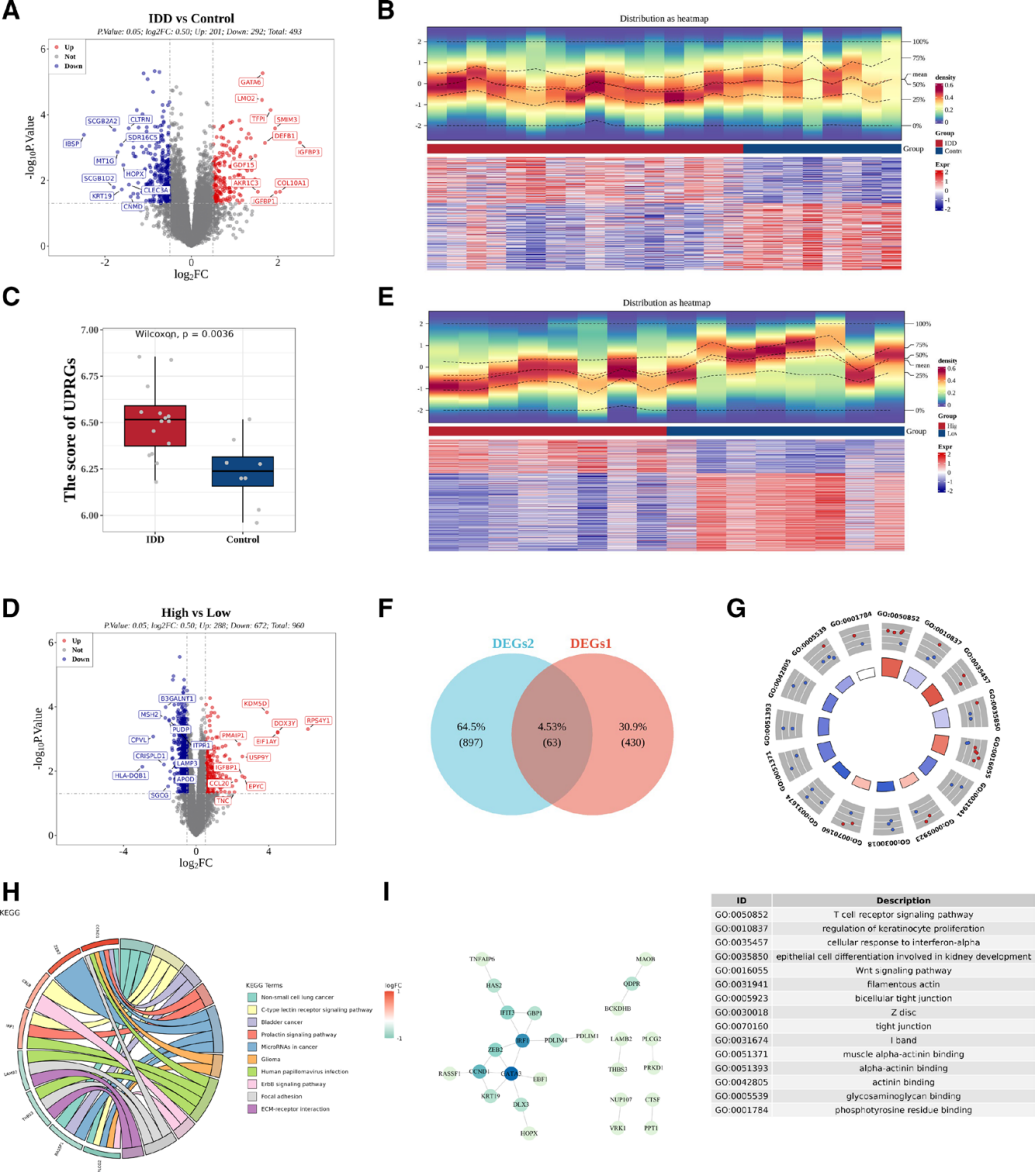
**Identification of UPRGs between IVDD and control samples within GSE70362 dataset.** (A) Volcano plot of the distribution of DEGs between IVDD and control samples within GSE70362 dataset. (B) Heatmap of differentially expressed genes. (C) Differences in ssGSEA scores for UPRGs. (D) Volcano plot of the distribution of differentially expressed genes between ssGSEA score groups with high and low UPRGs. (E) Heatmap of differentially expressed genes between groups of ssGSEA scores with high and low UPRGs. (F) Venn diagram of the intersection of DEGs1 and DEGs2. (G) Circle plot of GO enrichment results for candidate genes. (H) KEGG enrichment results and chord diagrams for candidate genes. (I) PPI plots of candidate genes. DEG = differentially expressed gene, GO = gene ontology, IVDD= intervertebral disc degeneration, KEGG= Kyoto Encyclopedia of genes and genomes, PPI = protein–protein interaction, ssGSEA = single sample gene set enrichment analysis, UPRG = unfolded protein response related genes.

To identify genes associated with IDD, differential expression analysis was performed on the GSE70362 dataset. Using thresholds of *P* < .05 and |log2FC| > 0.5, a total of 493 DEGs were identified, including 201 upregulated and 292 downregulated in IDD. Their distribution was visualized using a volcano plot (Fig. 1A) and a heatmap (Fig. 1B).

To further explore the relationship between UPRGs and IDD, analysis of the training set revealed 21 d-UPRGs (*P* < .05, Table S1, Supplemental Digital Content, https://links.lww.com/MD/R593). The ssGSEA score of UPRGs was calculated for all samples, showing a significant difference between groups (Fig. 1C). Based on the median ssGSEA score (6.516), IDD samples were divided into high- and low-score groups. Differential analysis between these groups identified 960 DEGs, including 288 upregulated and 672 downregulated (Fig. 1D, E).

The intersection of DEGs1 and DEGs2 yielded 63 candidate genes related to UPRGs (Fig. 1F). GO analysis revealed enrichment in 315 biology process, 29 cellular component, and 25 molecular function (*P* < .05). In biology process, candidate genes were mainly involved in TCR signaling, IFN-α response, and Wnt signaling; in cell component, they were associated with filamentous actin, bicellular tight junction, Z disc, tight junction, and I band; in molecular function, they were enriched in muscle α-actinin binding, α-actinin binding, actinin binding, glycosaminoglycan binding, and phosphotyrosine residue binding (Fig. 1G). KEGG analysis identified 10 enriched pathways, including C-type lectin receptor signaling, prolactin signaling, miRNA pathway, ErbB signaling, focal adhesion, and ECM–receptor interaction, etc (Fig. 1H).

The 63 candidate genes were uploaded to the STRING database to construct a PPI network (Fig. 1I). After removing isolated nodes, 26 hub genes were identified for further analysis: *PRKD1*, PLCG2, *PDLIM1*, *VRK1*, *NUP107*, *MAOB*, *THBS3*, *LAMB2*, *PDLIM4*, *TNFAIP6*, *HAS2*, *IFIT3*, *GBP1*, *IRF1*, *EBF1*, *HOPX*, *DLX3*, *PPT1*, *CTSF*, *RASSF1*, *GATA3*, *KRT19*, *ZEB2*, *CCND1*, *QDPR*, *BCKDHB*.

### 3.2. Two-sample MR analysis for candidate gene identification

To explore the potential causal relationship between core genes and IDD, we performed a two-sample MR analysis. The exposure data of 26 core genes were obtained from the eQTL GWAS datasets in the IEU OpenGWAS database, and the outcome data of IDD were extracted from the UK Biobank cohort (trait ID: ukb-b-19807), including 463,010 European individuals (1045 cases and 461,965 controls).

**Figure 2.**
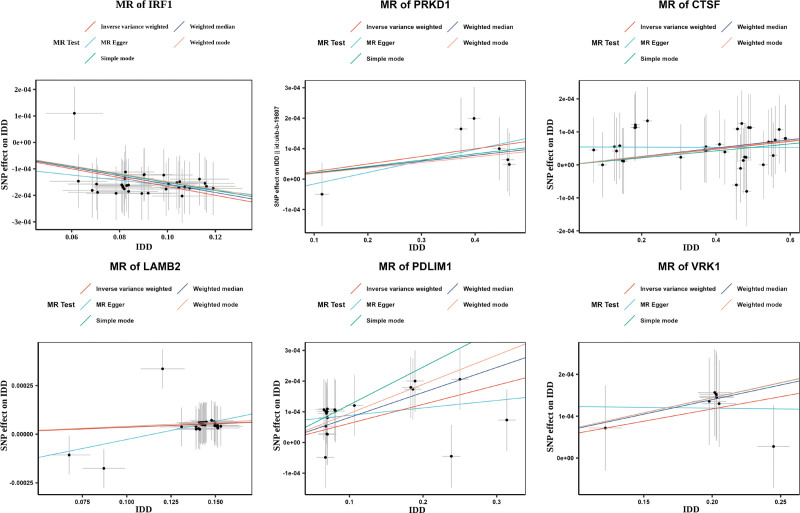
Scatter plots of two-sample MR analysis for core genes. IDD = intervertebral disc degeneration, MR = Mendelian randomization, SNP = single nucleotide polymorphisms.

**Figure 3.**
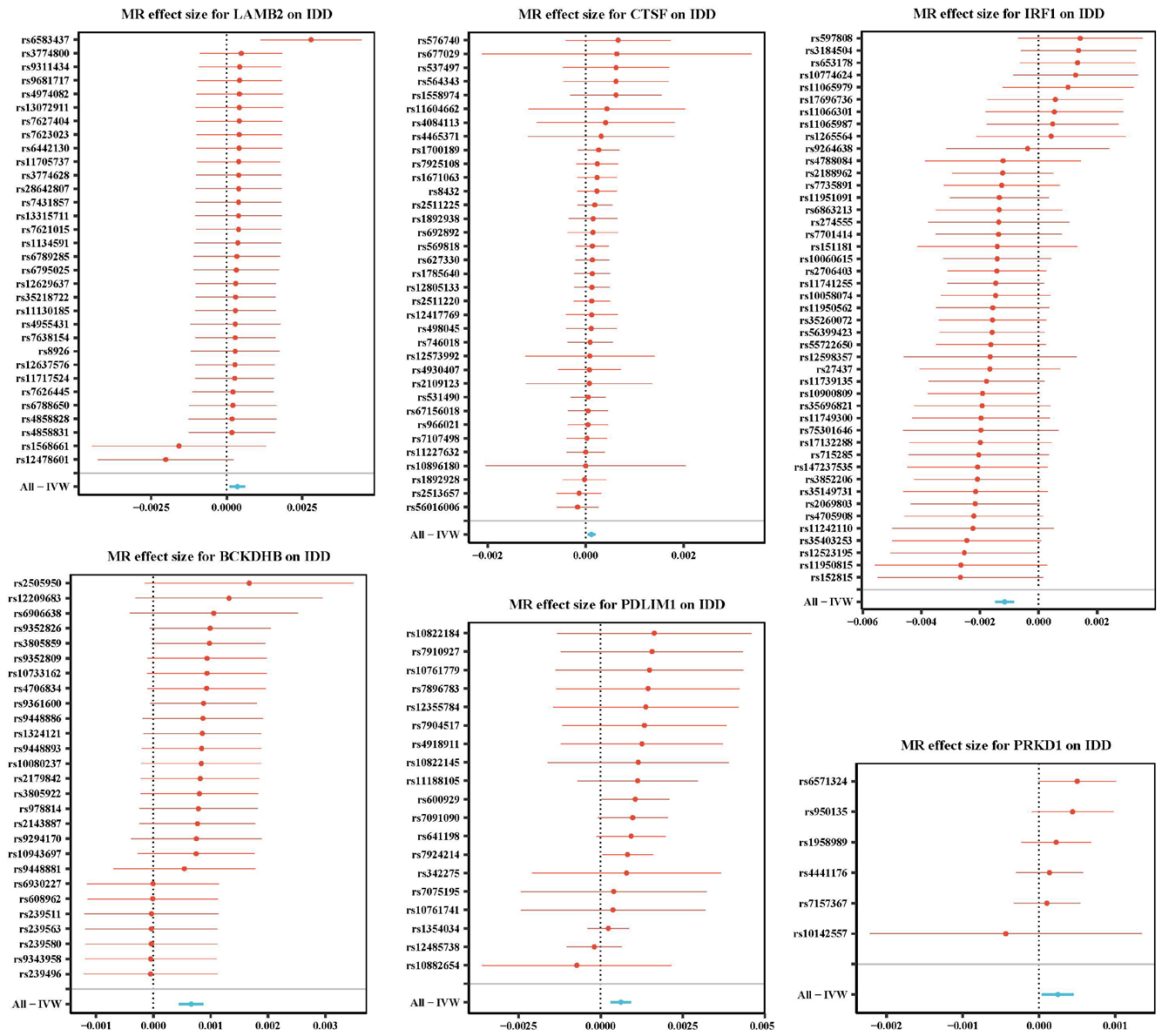
Forest plot of MR-estimated effects of core genes on IDD. IDD = Intervertebral disc degeneration, IVW = inverse variance weighted, MR = Mendelian randomization.

**Figure 4.**
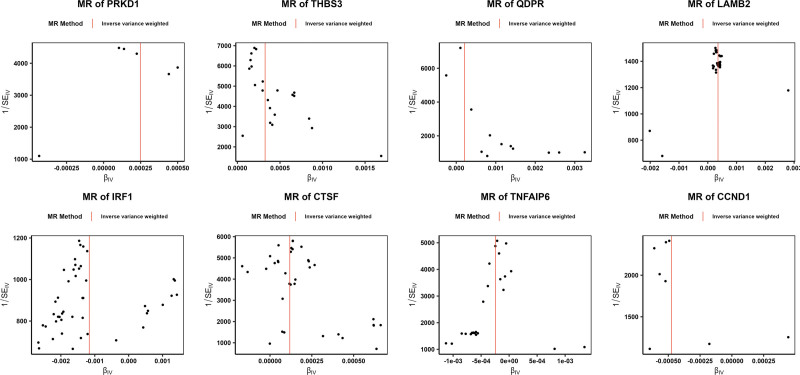
Funnel plots assessing horizontal pleiotropy in MR analysis. MR = Mendelian randomization.

After obtaining exposure data of 26 core genes and removing those with insufficient SNPs, 26 genes were included for MR analysis, with IDD as the outcome. IVW results showed that 11 genes were significantly associated with IDD (*P* < .05). Among them, the odds ratio (OR) values of *BCKDHB*, *VRK1*, *PDLIM1*, *QDPR*, *THBS3*, *LAMB2*, *CTSF*, and *PRKD1* were >1. The scatter plot showed that the IVW slope was positive and the intercept was close to 0 (Fig. 2, Figure S1A, Supplemental Digital Content, https://links.lww.com/MD/R593), and the forest plot revealed an overall effect >0 (Fig. 3, Figure S1B, Supplemental Digital Content, https://links.lww.com/MD/R593), suggesting that these genes were risk factors for IDD. Conversely, the OR values of *CCND1*, TNFAIP6, and *IRF1* were <1. The scatter plot showed a negative IVW slope, and the intercept was close to 0, and the forest plot revealed an overall effect <0, indicating that they were protective factors for IDD. All 11 genes passed the Steiger directionality test (Table 1). In addition, the funnel plots indicated that SNPs were symmetrically distributed around the IVW line, consistent with Mendel second law (Fig. 4, Figure S2A, Supplemental Digital Content, https://links.lww.com/MD/R593).

**Table 1:** MR analysis on significant causal exposure factors for IDD.

Id.exposure	Outcome	SYMBol	Nsnp	*P*-val	Or
eqtl-a-ENSG00000083123	Diagnoses - main ICD10: M51.3 other specified intervertebral disk degeneration ‖ id:ukb-b-19807	*BCKDHB*	26	5.18E-09	1.000649676
eqtl-a-ENSG00000100749		*VRK1*	8	.001226855	1.000586441
eqtl-a-ENSG00000107438		*PDLIM1*	13	5.04E-06	1.000907463
eqtl-a-ENSG00000110092		*CCND1*	10	.0033125	0.999502749
eqtl-a-ENSG00000123610		TNFAIP6	32	2.08E-05	0.999759703
eqtl-a-ENSG00000125347		*IRF1*	32	3.28E-18	0.99833767
eqtl-a-ENSG00000151552		*QDPR*	13	.032689119	1.000393398
eqtl-a-ENSG00000169231		*THBS3*	36	2.63E-24	1.000419232
eqtl-a-ENSG00000172037		*LAMB2*	34	.008040441	1.000324253
eqtl-a-ENSG00000174080		*CTSF*	39	.003341625	1.000112545
eqtl-a-ENSG00000184304		*PRKD1*	12	.007171858	1.000281145

IDD = intervertebral disc degeneration, MR = Mendelian randomization.

**Figure 5.**
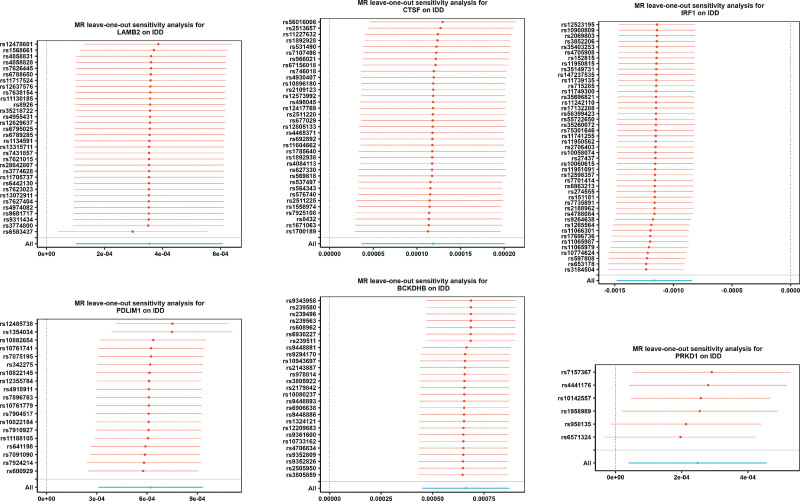
Leave-one-out sensitivity analysis for MR candidate genes. IDD = Intervertebral disc degeneration, MR = Mendelian randomization.

Further sensitivity analyses confirmed the robustness of the results. Heterogeneity testing indicated that *QDPR* had *Q*–*P*-val < .05 and was excluded, while pleiotropy testing identified TNFAIP6 and *THBS3* as potential confounding factors (*P* < .05), which were also excluded (Table 2, 3). Leave-one-out analysis showed that no single SNP significantly influenced the outcome (Fig. 5, Figure S2B, Supplemental Digital Content, https://links.lww.com/MD/R593). Collectively, these findings demonstrate the reliability and stability of the MR analysis. Therefore, 8 genes, namely *BCKDHB*, *VRK1*, *PDLIM1*, *CCND1*, *IRF1*, *LAMB2*, *CTSF*, and *PRKD1*, were identified as MR candidate genes for subsequent analyses.

**Table 2:** MR heterogeneity analysis for core genes and IDD.

Id.exposure	Outcome	SYMBol	*Q*	*Q*_df	*Q*_*P*-val
eqtl-a-ENSG00000083123	Diagnoses - main ICD10: M51.3 other specified intervertebral disk degeneration ‖ id:ukb-b-19807	*BCKDHB*	14.22322789	25	.957700439
eqtl-a-ENSG00000100749		*VRK1*	1.790628162	7	.970515127
eqtl-a-ENSG00000107438		*PDLIM1*	2.651032459	12	.997546298
eqtl-a-ENSG00000110092		*CCND1*	1.729894795	9	.995055091
eqtl-a-ENSG00000123610		TNFAIP6	17.87849283	31	.971075539
eqtl-a-ENSG00000125347		*IRF1*	8.253774218	31	.99998564
eqtl-a-ENSG00000151552		*QDPR*	27.80727074	12	.005902524
eqtl-a-ENSG00000169231		*THBS3*	46.92519149	35	.085764984
eqtl-a-ENSG00000172037		*LAMB2*	2.099329409	33	1
eqtl-a-ENSG00000174080		*CTSF*	12.0797864	38	.99998077
eqtl-a-ENSG00000184304		*PRKD1*	8.682299717	11	.651191071

IDD = intervertebral disc degeneration, MR = Mendelian randomization.

**Table 3:** Steiger directionality test for core genes and IDD.

Id.exposure	Outcome	SYMBol	Egger_intercept	SE	*P*-val
eqtl-a-ENSG00000083123	Diagnoses - main ICD10: M51.3 other specified intervertebral disk degeneration ‖ id:ukb-b-19807	*BCKDHB*	1.86E-05	0.000160748	.908745571
eqtl-a-ENSG00000100749		*VRK1*	0.000126756	0.000224755	.593209891
eqtl-a-ENSG00000107438		*PDLIM1*	−1.52E-05	5.90E-05	.800875728
eqtl-a-ENSG00000110092		*CCND1*	4.82E-05	9.07E-05	.609745613
eqtl-a-ENSG00000123610		TNFAIP6	−7.39E-05	3.60E-05	.049081427
eqtl-a-ENSG00000125347		*IRF1*	−6.47E-05	0.000103927	.538475659
eqtl-a-ENSG00000151552		*QDPR*	0.000152199	4.09E-05	.003394311
eqtl-a-ENSG00000169231		*THBS3*	0.000145042	6.65E-05	.036147454
eqtl-a-ENSG00000172037		*LAMB2*	−0.000202724	0.000171651	.24629522
eqtl-a-ENSG00000174080		*CTSF*	6.12E-05	4.05E-05	.1389487
eqtl-a-ENSG00000184304		*PRKD1*	8.98E-05	4.55E-05	.076815567

IDD = intervertebral disc degeneration, SE = standard error.

### 3.3. Identification of key genes IRF1 and PRKD1

**Figure 6.**
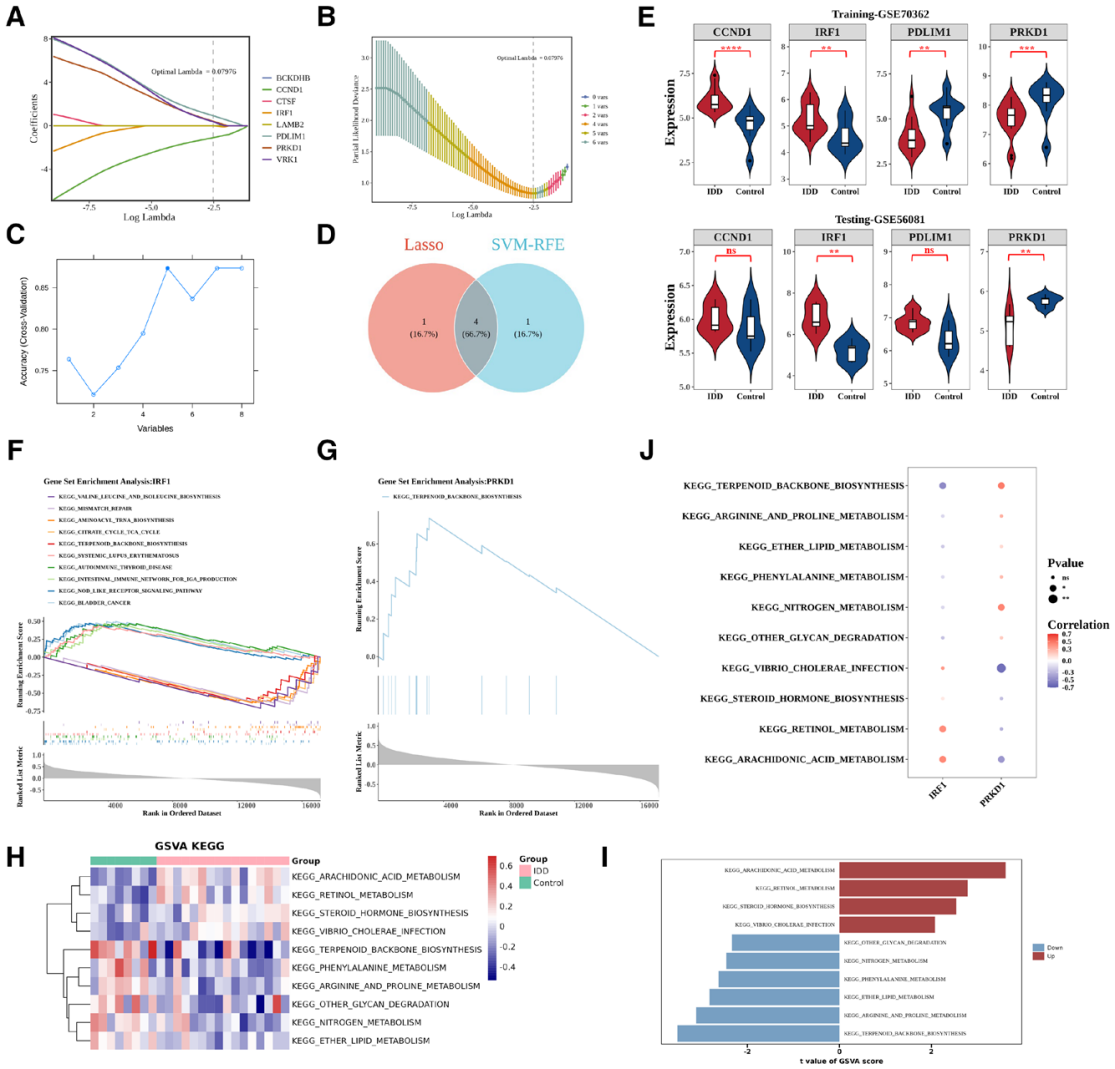
**Identification of key genes using machine learning and functional enrichment.** (A–B) LASSO regression analysis and selection of optimal lambda for feature gene screening. (C) SVM-RFE analysis showing the feature gene selection process. (D) Venn diagram displaying the intersection of genes identified by LASSO and SVM-RFE. (E) Expression patterns of key genes in GSE70362 and GSE56081 datasets. (F–G) GSEA enrichment plots showing top positively and negatively enriched pathways associated with *IRF1*and *PRKD1*. (H–I) GSVA-based KEGG pathway enrichment heatmaps comparing IDD and control groups. (J) Spearman correlation between key genes and differential KEGG pathways. GSEA = gene set enrichment analysis, GSVA = gene set variation analysis, IDD = intervertebral disc degeneration, KEGG = Kyoto Encyclopedia of genes and genomes, LASSO = Least absolute shrinkage and selection operator, SVM-RFE= support vector machine–recursive feature elimination, *P < .05; **P < .01; ***P < .001; ****P < .0001; ns indicates not significant.

Based on the expression profiles of the 8 MR candidate genes, 2 machine learning algorithms were applied to further screen potential feature genes. LASSO regression analysis identified 5 genes (*VRK1*, *PDLIM1*, *CCND1*, *IRF1*, and *PRKD1*) when the optimal lambda value was 0.07976 (Fig. 6A, B). Similarly, SVM-RFE achieved the highest prediction accuracy with 5 genes (*CCND1*, *BCKDHB*, *PRKD1*, *PDLIM1*, and *IRF1*) (Fig. 6C). The intersection of the 2 algorithms yielded 4 overlapping feature genes: *PDLIM1*, *CCND1*, *IRF1*, and *PRKD1* (Fig. 6D).

To validate their expression patterns, these 4 genes were examined in both the training set (GSE70362) and the validation set (GSE56081). *IRF1* expression was elevated in IDD, whereas *PRKD1* expression was reduced, and both genes showed consistent and significant differential expression across datasets (*P* < .05), thereby being identified as key genes for IDD (Fig. 6E).

### 3.4. Key genes were involved in complex functions and pathways

To investigate the biological functions of the key genes *IRF1* and *PRKD1*, GSEA and GSVA analyses were performed. For GSEA, Spearman correlation coefficients between each key gene and all other genes in the IDD samples of the training set were calculated to rank genes. *IRF1* was significantly enriched in 36 pathways, including bladder cancer, NOD-like receptor signaling, intestinal immune network for IgA production, autoimmune thyroid disease, systemic lupus erythematosus, TCA cycle, aminoacyl-tRNA biosynthesis, and branched-chain amino acid biosynthesis. *PRKD1* was enriched in terpenoid backbone biosynthesis. The top 5 positively and negatively enriched pathways for each gene were visualized (Fig. 6F, G).

GSVA was then applied to compare KEGG pathway activity between IDD and control groups. Ten differential pathways were identified (four upregulated and 6 downregulated), including terpenoid backbone biosynthesis, arginine and proline metabolism, ether lipid metabolism, phenylalanine metabolism, nitrogen metabolism, other glycan degradation, Vibrio cholerae infection, steroid hormone biosynthesis, retinol metabolism, and arachidonic acid metabolism (Fig. 6H, I).

Spearman correlation analysis between key genes and these differential pathways showed that *PRKD1* had the strongest positive correlation with terpenoid backbone biosynthesis (*R* = 0.4765) and the strongest negative correlation with Vibrio cholerae infection (*r* = −0.6104), indicating potential functional involvement in multiple IDD-related pathways (Fig. 6J).

### 3.5. Key genes modulate immune infiltration in IDD

**Figure 7.**
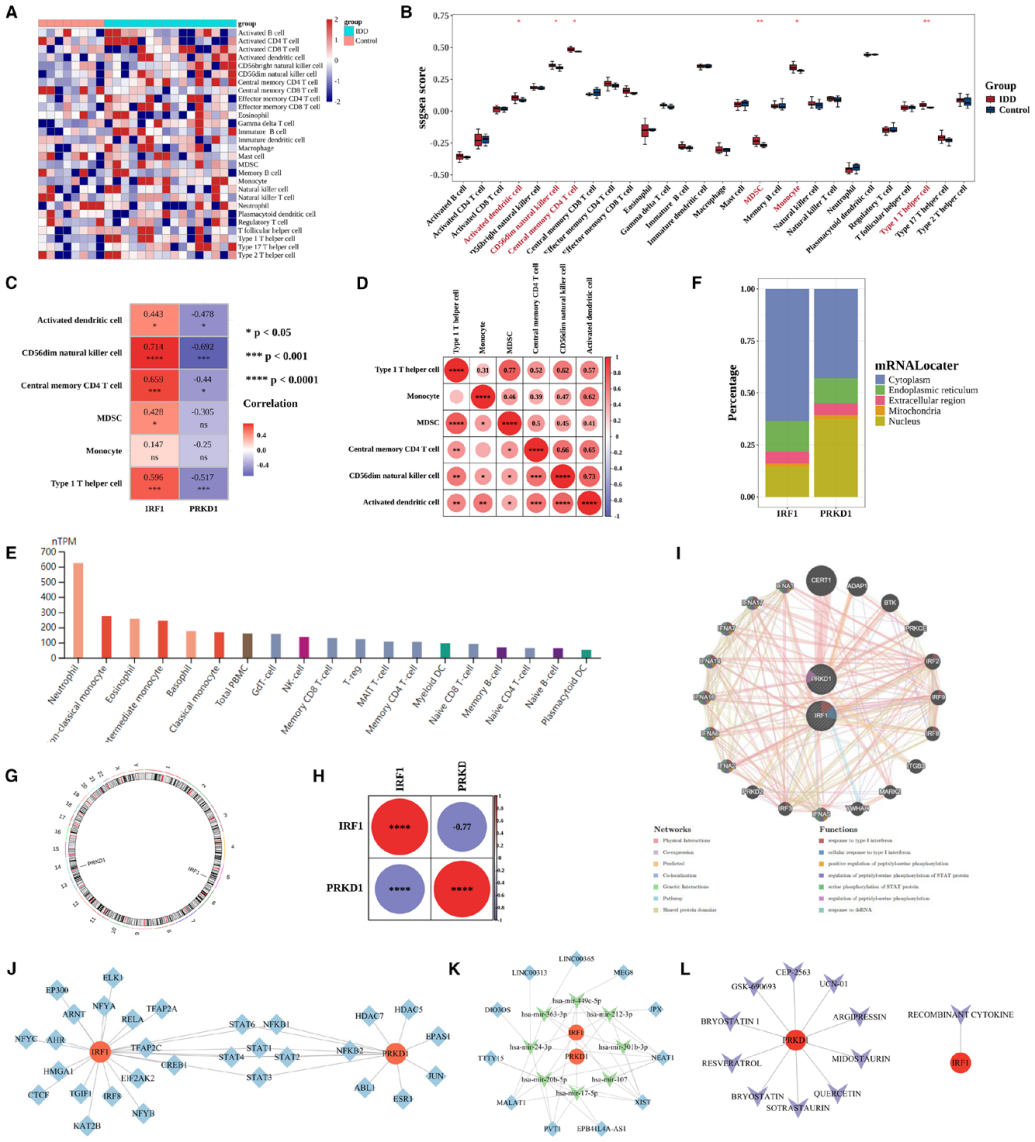
**Predictive analysis of key gene regulators Using public databases.** (A) Heatmap showing ssGSEA scores of 28 immune cell types across samples. (B) Boxplots comparing differential immune cell infiltration between IDD and control groups. (C) Correlation between key genes and 6 significantly altered immune cells. (D) Correlation heatmap among 6 differential immune cells. (E) Expression of key genes across immune cell types from the human protein atlas. (F) Predicted subcellular localization of *IRF1* and *PRKD1* using mRNALocater. (G) Chromosomal distribution of *IRF1* and *PRKD1* visualized using RCircos. (H) Correlation between *IRF1* and *PRKD1*. (I) Functional gene network predicted by GeneMANIA for *IRF1* and *PRKD1*. (J) Transcription factor–key gene regulatory network predicted using TRRUST. (K) lncRNA–miRNA–mRNA regulatory network of *IRF1* and *PRKD1*. (L) Predicted drug–gene interactions obtained from DGIdb. IDD = Intervertebral disc degeneration, lncRNA = long non-coding RNA, miRNA = microRNA, ssGSEA = single sample gene set enrichment analysis *P < .05; **P < .01; ***P < .001; ****P < .0001; ns indicates not significant.

We analyzed the role of key genes in the immune microenvironment of IDD. Firstly, the enrichment scores of 28 immune infiltrating cells were calculated using the ssGSEA algorithm based on the training set GEO data, and visualized in a heatmap (Fig. 7A). Box plots comparing the IDD and control groups revealed that 6 immune cell types: activated dendritic cell, CD56 dim natural killer cell, MDSC, monocyte, central memory CD4 T cell, and type 1 T helper cell: were significantly elevated in IDD samples (*P* < .05, Fig. 7B).

Correlation analysis among these 6 differential immune cells showed significant positive correlations with each other (Fig. 7D). Spearman correlation analysis between key genes and the 6 differential immune cells indicated that *IRF1* had the strongest positive correlation with CD56^dim natural killer cells (*R* = 0.714), while *PRKD1* showed the strongest negative correlation with CD56^dim natural killer cells (*r* = −0.692) (Fig. 7C). Correlation analysis among the 6 differential immune cells and corresponding heatmap demonstrated that MDSC and Type 1 T helper cell had the strongest positive correlation (*R* = 0.77) (Fig. 7D).

Furthermore, expression analysis based on the human protein atlas database revealed that *IRF1* was highly expressed in neutrophils, whereas *PRKD1* expression was not available in this dataset (Fig. 7E). These results suggest that key genes, particularly *IRF1* and *PRKD1*, may play a regulatory role in the immune microenvironment of IDD.

### 3.6. Subcellular and chromosomal localization of key genes

The FASTA sequences of the 2 key genes (*IRF1*, *PRKD1*) were retrieved from NCBI and input into the mRNALocater database to predict subcellular localization. Predicted scores across 5 compartments were displayed in stacked histograms, showing that both *IRF1* and *PRKD1* had the highest scores in the cytoplasm, indicating cytoplasmic localization (Fig. 7F). Chromosomal distribution of the key genes was visualized using the R package “RCircos,” revealing that *IRF1* and *PRKD1* were located on chromosomes 5 and 14, respectively (Fig. 7G).

### 3.7. The complex regulatory mechanisms of key genes and drugs prediction

Correlation analysis revealed a strong negative correlation between *IRF1* and *PRKD1 (r* = −0.77) (Fig. 7H). Subsequently, the GeneMANIA database was used to predict genes functionally related to *IRF1* and *PRKD1*, including *CERT1*, *ADAP1*, and *BTK*, which were mainly involved in type I interferon (IFN) response, peptidyl-serine phosphorylation, STAT protein phosphorylation, and dsRNA response (Fig. 7I). Furthermore, TFs targeting the key genes were predicted using the TRRUST database, and a regulatory network comprising 32 nodes and 36 edges was constructed. Among them, *STAT2*, *STAT6*, *NFKB1*, *STAT1*, *STAT3*, and *STAT4* were identified as common regulators of both genes (Fig. 7J). Using the NetworkAnalyst platform, miRNAs targeting *IRF1* and *PRKD1* were predicted, and the corresponding lncRNAs were obtained from the miRNet, Starbase, and miRcode2 databases. A total of 11 intersecting lncRNAs were identified, leading to the construction of an lncRNA–miRNA–mRNA regulatory network containing 2 mRNAs, 8 miRNAs, and 11 lncRNAs (Fig. 7K). Finally, drug prediction was performed using the DGIdb database, which identified 1 drug targeting *IRF1* and 10 drugs targeting *PRKD1* (Fig. 7L).

### 3.8. Single-cell expression analysis of key genes

**Figure 8.**
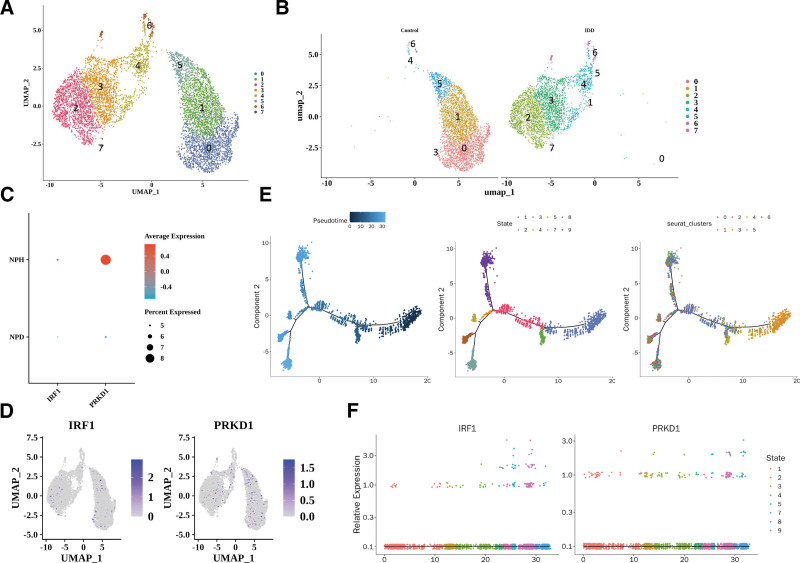
**Single-cell RNA sequencing analysis of key genes in NP tissues.** (A) UMAP visualization of cell populations in control and IDD NP samples. (B) Clustering patterns highlighting differences between control and IDD groups. (C) Expression of *IRF1* and *PRKD1* across individual cells. (D) UMAP plots showing the spatial distribution of *IRF1* and *PRKD1* in cell clusters. (E) Pseudotime trajectory reconstruction of NP cells along IDD progression. (F) Expression dynamics of *IRF1* and *PRKD1* along pseudotime trajectory, showing *IRF1* upregulation and *PRKD1* downregulation with disease progression. IDD = Intervertebral disc degeneration, NP = nucleus pulposus, UMAP = uniform manifold approximation and projection.

To further investigate the expression patterns of *IRF1* and *PRKD1* at the single-cell level, the scRNA-seq dataset GSE199866 was analyzed. After quality control and filtering, a total of 7633 high-quality cells and 19,284 genes were retained for downstream analysis (Figure S3, Supplemental Digital Content, https://links.lww.com/MD/R593). The UMAP visualization revealed a clear distribution of cell populations (Fig. 8A), and further stratification showed distinct clustering patterns between the control and IDD groups (Fig. 8B). The expression analysis demonstrated that *IRF1* was significantly upregulated in IDD samples, whereas *PRKD1* showed a marked decrease (Fig. 8C). The UMAP plots further confirmed the differential spatial distribution of *IRF1* and *PRKD1* expression across cell clusters (Fig. 8D).

To explore the temporal dynamics, pseudotime trajectory analysis was performed. The global trajectory indicated progressive transcriptional changes along the IDD process (Fig. 8E). Specifically, *IRF1* expression increased with disease progression, showing the highest expression at late stages, while *PRKD1* expression gradually decreased along the trajectory (Fig. 8F). Collectively, these findings suggest that *IRF1* and *PRKD1* exhibit opposite expression dynamics during IDD progression, with *IRF1* potentially promoting disease exacerbation and *PRKD1* playing a protective role.

## 4. Discussion

In this study, we systematically explored the molecular landscape of IDD and its association with UPR by integrating multiple bioinformatics approaches. Starting with transcriptome data from public GEO datasets, we performed DEG analysis, ssGSEA-based scoring, functional enrichment, and PPI network construction to identify candidate genes associated with IDD and UPRGs. Two-sample MR further clarified causal relationships, leading to 8 MR candidate genes. ML algorithms (LASSO and SVM-RFE) refined the candidates to 4 feature genes, of which *IRF1* and *PRKD1* were consistently differentially expressed across training and validation datasets. Subsequent functional analyses, including GSEA, GSVA, immune infiltration, scRNA-seq profiling, subcellular and chromosomal localization, and regulatory network construction, revealed that these 2 key genes are involved in immune modulation, IFN signaling, STAT protein phosphorylation, and complex lncRNA–miRNA–mRNA interactions. Drug prediction highlighted potential therapeutic targets. Collectively, our findings identify *IRF1* and *PRKD1* as key regulators in IDD pathogenesis, providing novel insights into disease mechanisms and potential intervention strategies, representing a comprehensive integration of multi-omics analyses and single-cell validation in the study of IDD.

In the training set GSE70362, which included 24 samples (12 healthy controls and 12 IDD patients), differential expression analysis identified 493 DEGs associated with IDD. To investigate UPR involvement, these DEGs were intersected with UPR-related genes, and ssGSEA was applied to calculate UPR enrichment scores for each sample, dividing them into high- and low-score groups. Differential analysis between these groups yielded 960 UPR-related DEGs. GO and KEGG enrichment analyses using the “clusterProfiler” R package revealed that these genes were mainly enriched in pathways related to TCR signaling, IFN-α response, Wnt signaling, apoptosis, and ECM–receptor interaction, etc A PPI network was constructed using the 63 candidate genes in the STRING database, and isolated nodes were removed. Based on network topology metrics such as degree centrality, 26 hub genes with the highest connectivity and potential functional importance were selected for subsequent analyses.

To further clarify the causal associations between candidate genes and IDD, we applied two-sample MR analysis, an analytical framework that leverages genetic variants as instrumental variables to infer causal relationships between exposures and outcomes. This approach has been widely used in complex diseases to overcome confounding and reverse causation, and has demonstrated robust value in uncovering disease-related biomarkers and therapeutic targets in musculoskeletal and degenerative disorders.^[25–27]^ These 26 hub genes were then subjected to two-sample MR, which identified 11 MR candidate genes with statistically significant causal effects on IDD. Among these, *BCKDHB*, *VRK1*, *PDLIM1*, *QDPR*, *THBS3*, *LAMB2*, *CTSF*, and *PRKD1* were identified as risk factors (OR > 1), suggesting that their upregulation may accelerate disc degeneration, while *CCND1*, TNFAIP6, and *IRF1* were identified as protective factors (OR < 1), potentially contributing to resistance against degenerative processes. Forest plots were generated to visualize the effect sizes and confidence intervals of each genetic instrument, facilitating the assessment of consistency across SNPs. The Steiger directionality test was applied to confirm that the genetic variants influenced IDD through the exposure genes rather than vice versa, thereby strengthening causal directionality. Finally, leave-one-out analysis was conducted to evaluate the influence of individual SNPs on the overall estimates, demonstrating that no single variant disproportionately drove the results. Collectively, these rigorous methodological layers provide strong evidence that the identified genes exert divergent causal roles in IDD pathogenesis as either protective or risk contributors.

To further refine the candidate genes identified by two-sample MR, we incorporated machine learning approaches, which have emerged as powerful tools for high-dimensional biological data analysis and biomarker discovery.^[28–30]^ Unlike conventional statistical methods, machine learning can capture complex, nonlinear relationships in gene expression profiles, thereby improving the accuracy of feature selection and disease prediction. In this study, two complementary machine learning algorithms were applied: LASSO regression and SVM-RFE. LASSO regression selects key disease-associated genes while avoiding overfitting, whereas SVM-RFE iteratively optimizes predictive features, together providing robust screening in small-sample, high-dimensional datasets. LASSO regression identified *VRK1*, *PDLIM1*, *CCND1*, *IRF1*, and *PRKD1* as potential markers, while SVM-RFE achieved the highest prediction accuracy with *CCND1*, *BCKDHB*, *PRKD1*, *PDLIM1*, and *IRF1*. The intersection of these 2 methods yielded 4 overlapping feature genes: *PDLIM1*, *CCND1*, *IRF1*, and *PRKD1*. To validate their reliability, we examined their expression across both the training set (GSE70362) and the validation set (GSE56081). Among them, *IRF1* expression was consistently elevated in IDD, whereas *PRKD1* expression was consistently decreased, and both showed significant differential expression across datasets (*P* < .05). Therefore, *IRF1* and *PRKD1* were ultimately identified as the key genes most robustly associated with IDD pathogenesis.

*IRF1*, IFN regulatory factor 1, is an important TF whose molecular localization is mainly in the nucleus and which plays a regulatory role by binding to the promoter region of target genes.^[31–33]^ Functionally, *IRF1* is involved in immune response, inflammation, apoptosis, and cell cycle regulation, inducing the expression of cytokines such as IFN and mediating cell growth inhibition and apoptosis signals.^[34–36]^ In pathway regulation, *IRF1* is a key molecule in the IFN signaling pathway, which can be activated by the JAK-STAT pathway; meanwhile, *IRF1* cross-regulates with the TLR (toll-like receptor) pathway and the NF-κB pathway, and jointly mediates inflammatory and immune responses.^[37,38]^ In degenerative diseases, *IRF1* is involved in the pathological process of Alzheimer disease, Parkinson disease, osteoarthritis, etc by promoting the release of pro-inflammatory factors (e.g., TNF-α, IL-6) and apoptosis, which exacerbate the tissue damage and functional deterioration.^[39,40]^ Importantly, a recent study has shown that the programmed cell death of NP cells is closely associated with *IRF1* expression, and elevated *IRF1* promotes NPC apoptosis and inflammatory responses, contributing to extracellular matrix degradation and IDD.^[41,42]^

*PRKD1* (protein kinase D1) is a serine/threonine kinase that is mainly located in the cytoplasm of the cell, and upon activation, it can translocate to the cell membrane, Golgi apparatus, or nucleus to transmit signals by phosphorylating target proteins.^[43]^ PRKD has a wide range of functions, including the regulation of cell proliferation, apoptosis, differentiation, cytoskeletal remodeling, and vesicular transport, as well as the maintenance of intracellular homeostasis and stress responses.^[44,45]^ At the pathway-level, *PRKD1* is often activated by the protein kinase C family, which in turn participates in the regulation of MAPK and NF-κB pathways, and also affects actin reorganization to correlate with cell polarity and migration.^[46,47]^ In degenerative diseases, *PRKD1* has been reported to be associated with neurodegenerative diseases (e.g., Alzheimer disease, possibly through regulation of tau protein phosphorylation or neuroinflammation), osteoarthritis (affecting chondrocyte metabolism and matrix homeostasis), etc, and its aberrant activation or expression may exacerbate tissue degeneration.^[48,49]^ In response to IDD, *PRKD1* may be involved in the pathological process of intervertebral disc structural damage and functional decline by inhibiting NP cell survival, promoting the release of pro-inflammatory factors (e.g., IL-1β, TNF-α), and accelerating the degradation of extracellular matrix by up-regulating the expression of matrix metalloproteinases This is the first report of a possible study of *PRKD1* as a biomarker of IDD.

To further explore the functional roles of *IRF1* and *PRKD1* in IDD, GSEA and GSVA analyses were performed. GSEA revealed that *IRF1* was significantly enriched in 36 pathways, including NOD-like receptor signaling, intestinal immune network for IgA production, autoimmune thyroid disease, systemic lupus erythematosus, TCA cycle, aminoacyl-tRNA biosynthesis, and branched-chain amino acid biosynthesis, suggesting its broad involvement in immune regulation, inflammation, and cellular metabolism. The association of *IRF1* with inflammation-related pathways, such as NOD-like receptor signaling, characterized by cytokine activation, macrophage recruitment, and upregulation of catabolic genes, links UPR dysregulation to immune activation and subsequent extracellular matrix degradation, thereby establishing a functional bridge to disc structural deterioration. By contrast, *PRKD1* was mainly enriched in terpenoid backbone biosynthesis, indicating a more focused role in metabolic processes. GSVA further identified ten differential KEGG pathways between IDD and control groups, including terpenoid backbone biosynthesis, arginine and proline metabolism, ether lipid metabolism, phenylalanine metabolism, nitrogen metabolism, other glycan degradation, Vibrio cholerae infection, steroid hormone biosynthesis, retinol metabolism, and arachidonic acid metabolism. Spearman correlation analysis highlighted that *PRKD1* exhibited the strongest positive correlation with terpenoid backbone biosynthesis and the strongest negative correlation with Vibrio cholerae infection, suggesting that *PRKD1* may modulate specific metabolic and immune-related pathways in IDD. The enrichment of *PRKD1* in terpenoid backbone biosynthesis suggests its potential link to lipid metabolic stress, where disrupted lipid homeostasis can induce oxidative stress, mitochondrial dysfunction, and impaired extracellular matrix maintenance in NP cells, with its downregulation potentially compromising protective metabolic responses. Collectively, these results indicate that *IRF1* and *PRKD1* participate in complex and partly opposing molecular networks, potentially contributing to IDD pathogenesis through distinct functional mechanisms.

Our analyses further revealed that *IRF1* and *PRKD1* may play crucial roles in modulating the immune microenvironment of IDD. ssGSEA-based immune infiltration analysis showed that 6 immune cell types, including activated dendritic cells, CD56^dim natural killer cells, MDSCs, monocytes, central memory CD4 T cells, and type 1 T helper cells, were significantly elevated in IDD samples. Notably, *IRF1* expression exhibited a strong positive correlation with CD56^dim natural killer cells (*R* = 0.714), whereas *PRKD1* displayed a strong negative correlation (*r* = −0.692), indicating that these key genes may have opposing regulatory effects on immune cell populations. Further correlation analysis highlighted that MDSCs and type 1 T helper cells were positively interrelated, suggesting coordinated immune responses during disc degeneration. Subcellular localization predictions indicated that both *IRF1* and *PRKD1* are primarily cytoplasmic, while chromosomal mapping placed them on chromosomes 5 and 14, respectively. Regulatory network analyses revealed a complex interplay involving TFs (STAT1/2/3/4, STAT6, NFKB1), miRNAs, and lncRNAs, reflecting multilayered posttranscriptional and transcriptional control. Additionally, drug prediction identified several potential compounds targeting *IRF1* and *PRKD1*, providing avenues for therapeutic intervention. Drug–gene interaction analysis identified one compound targeting *IRF1* (recombinant cytokine) and ten compounds targeting *PRKD1*, including kinase modulators such as GSK-690693, midostaurin, CEP-2536, and sotrastaurin, as well as natural molecules like quercetin and resveratrol. These agents have documented roles in regulating inflammatory or stress-response pathways, suggesting potential applicability in modulating *IRF1*- or *PRKD1*-mediated mechanisms in IDD. Although preliminary, these predicted compounds provide a valuable starting point, and their therapeutic effects will be systematically evaluated in future cellular and animal studies. Single-cell transcriptomic analysis using GSE199866 further confirmed the differential expression patterns of these genes at the cellular level, with *IRF1* markedly upregulated and *PRKD1* downregulated in IDD, supporting their involvement in disease-specific immune and cellular processes. Collectively, these findings suggest that *IRF1* and *PRKD1* not only modulate immune infiltration but also participate in complex regulatory networks that may influence IDD progression at multiple cellular levels.

Despite the strengths of our integrative multi-omics and MR design, several limitations should be acknowledged. First, although GSE70362 provided an adequate sample size for DEG identification, the GSE56081 validation dataset contained only 5 IDD and 5 control samples, and was therefore used solely as an independent verification cohort; however, its small size may still limit the stability of external validation. Second, the UK Biobank GWAS used for MR included 1045 IDD cases among >460,000 participants, which, while enabling stringent IV selection and robust sensitivity analyses, may reduce statistical power to detect modest causal effects. The consistent causal associations for *IRF1* and *PRKD1* across multiple MR estimators mitigate concerns regarding underpowering, yet replication using larger GWAS datasets, such as FinnGen or future multi-ethnic cohorts, will be essential to strengthen generalizability. In addition, the eQTL data used in MR were derived primarily from whole blood within IEU OpenGWAS, which may not fully reflect transcriptional regulation in disc-resident cells, as UPR activation and endoplasmic reticulum-stress responses are highly tissue-specific. This mismatch is an inherent constraint given the absence of disc-specific eQTL datasets in current resources such as GTEx. Thus, future MR studies incorporating spine- or disc-specific eQTL data, when available, will be critical to refine the causal effects observed here. Together, these factors highlight the need for larger transcriptomic datasets and more physiologically relevant genetic resources in future investigations.

In summary, this study systematically evaluated the diagnostic and functional relevance of potential key genes in IDD using comprehensive bioinformatics approaches, including UPR-based analyses and two-sample MR. Among them, *IRF1* and *PRKD1* were identified as pivotal genes, and their associated molecular mechanisms were explored, providing novel insights for potential therapeutic strategies.

## 5. Conclusions

In conclusion, our study identified *IRF1* and *PRKD1* as key genes associated with IDD through integrated bioinformatics analyses, including UPR-based scoring and two-sample MR. Their expression patterns, functional pathways, immune regulatory roles, and potential molecular mechanisms were characterized, highlighting their relevance in IDD pathogenesis. These findings offer valuable insights for the development of novel diagnostic biomarkers and therapeutic strategies.

## Author contributions

**Conceptualization:** Feng Zheng, Zhilei Hu, Menglin Luo, Chenhao Liu.

**Formal analysis:** Feng Zheng, Jiawei Fu, Chao Liu.

**Funding acquisition:** Feng Zheng, Chenhao Liu.

**Methodology:** Feng Zheng, Zhilei Hu, Wenbo Yue, Yulin Ma.

**Visualization:** Feng Zheng, Menglin Luo, Wenbo Yue, Chao Liu.

**Data curation:** Jiawei Fu, Chao Liu.

**Project administration:** Jiawei Fu, Menglin Luo.

**Validation:** Jiawei Fu, Zhilei Hu, Wenbo Yue, Chao Liu.

**Software:** Zhilei Hu, Wenbo Yue, Yulin Ma.

**Supervision:** Zhilei Hu, Menglin Luo, Yulin Ma, Chenhao Liu.

**Resources:** Chao Liu.

**Investigation:** Chenhao Liu.

**Writing – original draft:** Feng Zheng, Zhilei Hu, Yulin Ma.

**Writing – review & editing:** Jiawei Fu, Chenhao Liu.

## Supplementary Material


