# Supplementary material for: Transcriptome data combined with two-sample Mendelian randomization reveal IRF1 and PRKD1 as UPR-related key regulators in intervertebral disc degeneration
Source: Medicine (Baltimore). 2026 Apr 3;105(14):e48213. doi: 10.1097/MD.0000000000048213 (PMC13052935; doi:10.1097/MD.0000000000048213)

**Supplementary Table and Figures**

**Supplementary Table1. Differential unfolded protein response -related genes**

| **UPRGs_DEG_genes** | **pvalue** |
| --- | --- |
| AMFR | 9.2E-17 |
| ATF3 | 1.9E-10 |
| CCND1 | 9.8E-42 |
| CREBZF | 0.00375 |
| DERL1 | 4.4E-16 |
| DNAJC10 | 0.00134 |
| ERN2 | 3E-61 |
| HSF1 | 5.1E-18 |
| NCK2 | 8.8E-06 |
| PACRG | 2.4E-07 |
| QRICH1 | 1.8E-09 |
| SERP2 | 1.5E-06 |
| TMED2 | 9.1E-24 |
| VAPB | 1.3E-18 |
| XBP1 | 4.2E-06 |
| ERN2 | 0.00045 |
| HSPA5 | 2.7E-05 |
| NCK1 | 3.3E-48 |
| PTPN1 | 1.1E-17 |
| RNF7 | 1.3E-19 |

**Supplementary Figure 1. Scatter plots and Forest plot.** (A) Scatter plots of two-sample MR analysis for core genes. (B) Forest plot of MR-estimated effects of core genes on IDD.


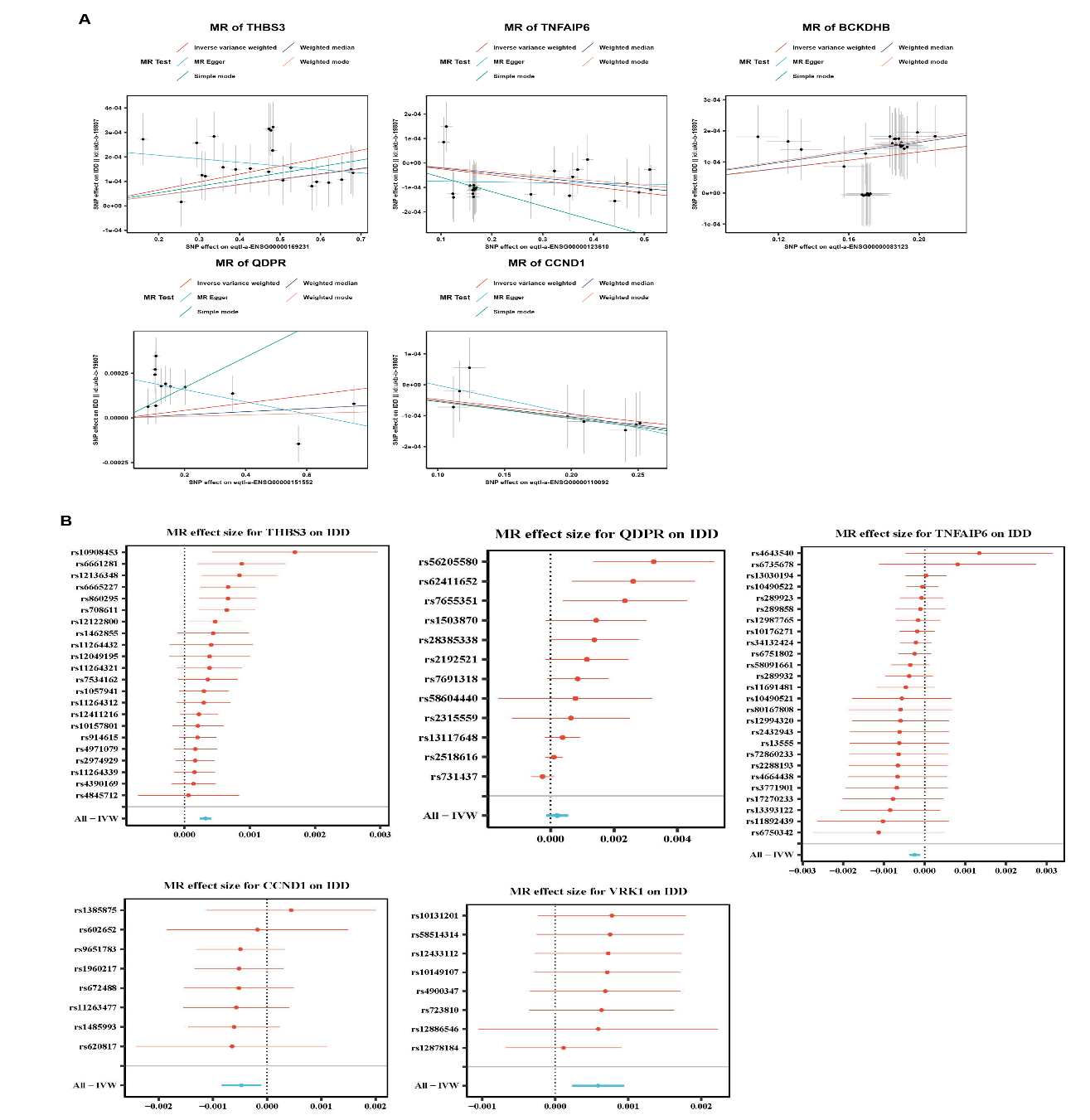


**Supplementary Figure 2. Horizontal pleiotropy.** (A) Funnel plots assessing horizontal pleiotropy in MR analysis. (B) Leave-one-out sensitivity analysis for MR candidate genes.


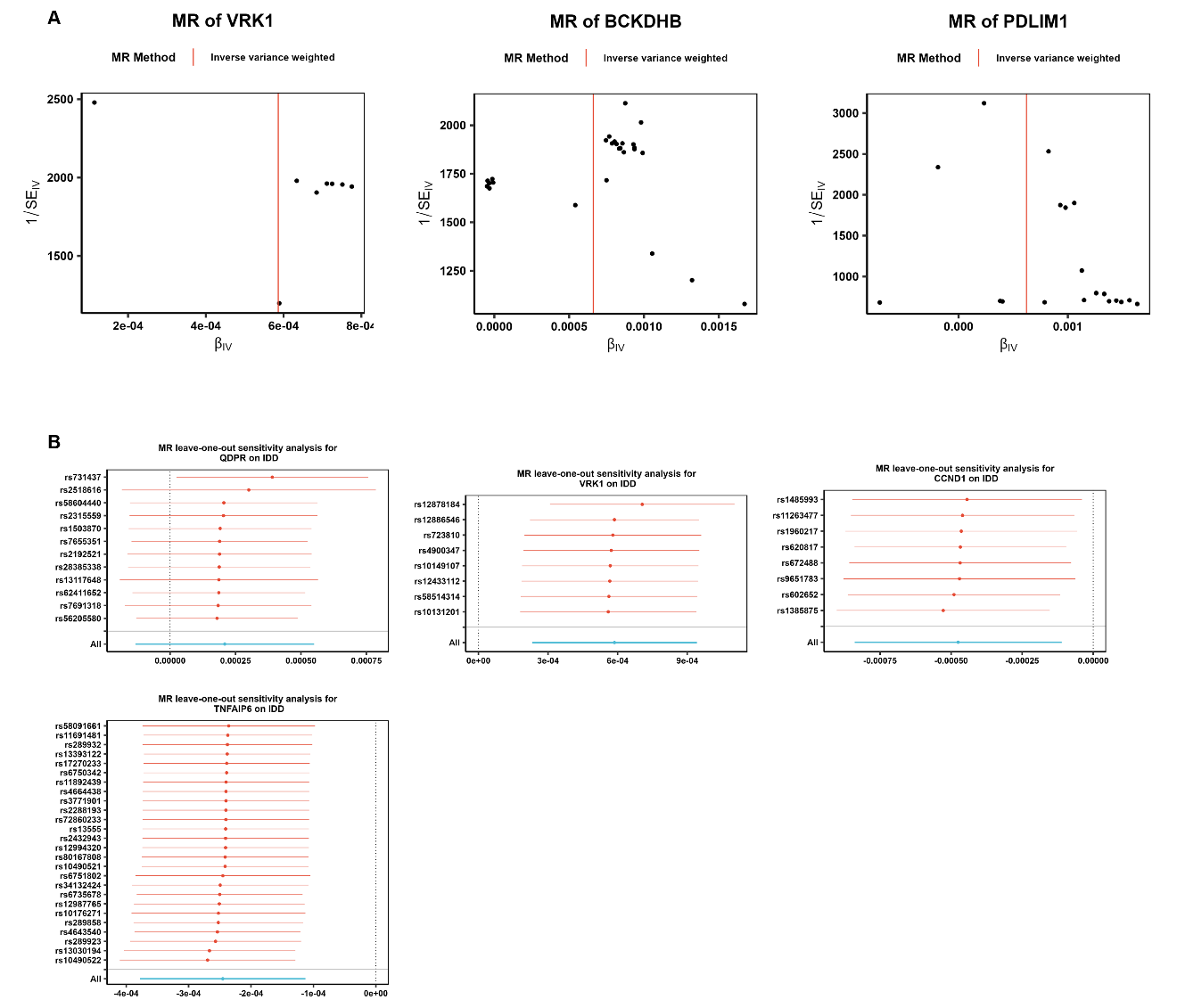


**Supplementary Figure 3. Single-cell RNA-sequencing (scRNA-seq) analysis of the GSE199866 dataset.** (A-B) Violin plots showing distributions of gene counts, total mRNA molecules and mitochondrial gene expression before and after quality control filtering. (C) Highly variable gene screen. (D) Principal Component Fractal Map. (E) Principal component line graph.


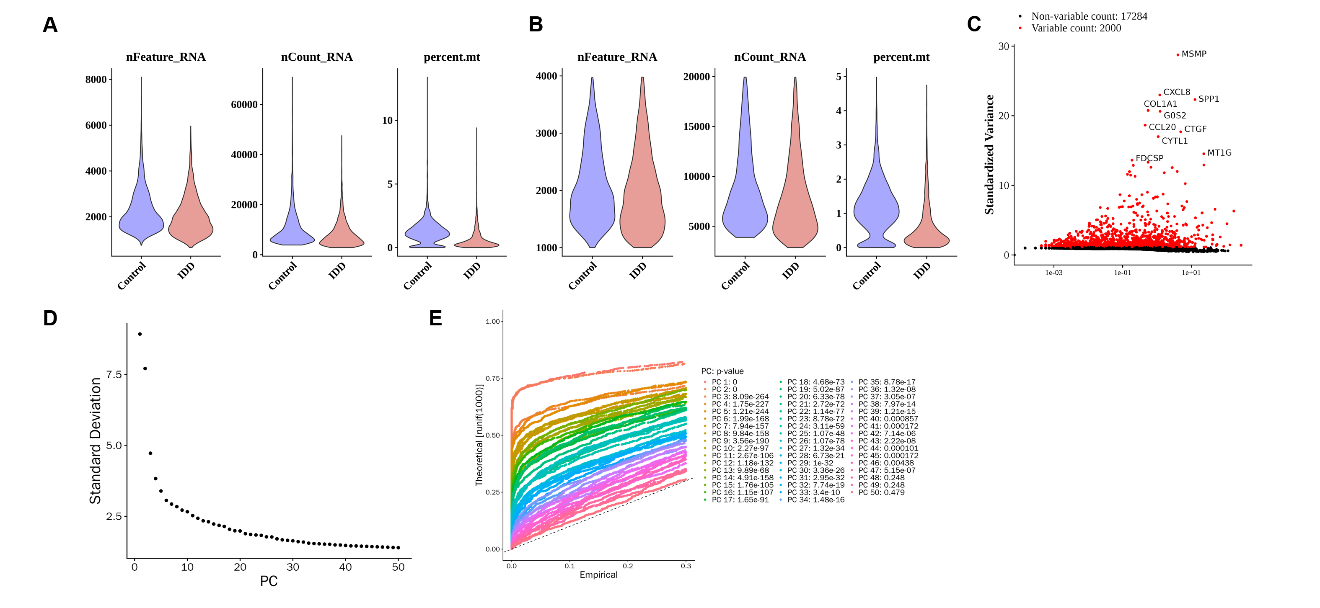

Supplement: Supplementary file 1 [file medi-105-e48213-s001.docx]
